# Surface-enhanced Raman scattering (SERS)–based immunosystem for ultrasensitive detection of the 90K biomarker

**DOI:** 10.1007/s00216-020-02903-2

**Published:** 2020-09-02

**Authors:** Valentina Gallo, Antonia Lai, Alessandra Pasquo, Salvatore Almaviva, Stefano Iacobelli, Luca Persichetti, Giovanni Capellini, Giovanni Antonini

**Affiliations:** 1 Interuniversity Consortium INBB - Biostructures and Biosystems National Institute, 00136 Rome, Italy; 2 ENEA, Via Enrico Fermi 45, 00044 Frascati, Italy; 3 Mediapharma srl, 66100 Chieti, Italy; 4 Department of Sciences, University Roma Tre, Viale G. Marconi 446, 00146 Rome, Italy

**Keywords:** Surface-enhanced Raman spectroscopy (SERS), SERS substrates, Biomarkers, LGAL3BP, Bioanalytical methods

## Abstract

The research and the individuation of tumour markers in biological fluids are currently one of the main tools to support diagnosis, prognosis, and monitoring of the therapeutic response in oncology. Although the identification of tumour markers in asymptomatic patients is crucial for early diagnosis, its application is still limited by the relatively low sensitivity and the complexity of existing methods (i.e. ELISA, mass spectrometry). We developed an easy, fast, and ultrasensitive surface-enhanced Raman scattering (SERS)–based system, for the detection and quantitation of the LGALS3BP (90K) biomarker that was used as a model, based on the development of antibody-functionalized nanostructured gold surfaces. The detection system was effective for the ultrasensitive detection and characterization of samples of different biochemical compositions. In conclusion, this work could provide the foundation for the development of a medical diagnostic device with the highest predictive power when compared with the methods currently used in cancer diagnostics.

**Electronic supplementary material:**

The online version of this article (10.1007/s00216-020-02903-2) contains supplementary material, which is available to authorized users.

## Introduction

The importance of early diagnosis of tumours lies on the possibility to detect cancer before the manifestation of clinical symptoms, allowing for prompt cures. Indeed, several cancers become apparent only at advanced stages [1, 2]. The research of the last decades has been focussed on the possibility to study cancer at a molecular level, and considerable progresses in this sense have been made: DNA microarray, DNA sequencing, multiplex PCR, and other genomic technologies have been successfully used to study molecular alterations in tumours through DNA copy number assessment [3], mutation screening, and gene expression and microRNA expression profiling [4]. In parallel with genomic tools, proteomics and metabolomics advances are opening the strongly impacting prospect of individuating and detecting the biochemical labels of cancer, or tumour markers: molecules (e.g. proteins, hormones, enzymes, peptides) that could be present in tissues and/or in body fluids of oncologic patients as a consequence of tumourigenesis and progression, representing unequivocal tumour signs [5, 6]. The finding and identification of tumour markers with predictive value represents an intriguing and promising possibility to detect and characterize cancer at its early stages [7–9]. Interestingly, the identification of biomarkers in biological fluids will repeal the invasiveness of solid tissue analysis. So far, several serum and plasma tumour markers have been identified and associated with different cancer types to monitor the cancer status and the response to therapies, including prostate-specific antigen for prostate cancer, CA 125 for ovarian cancer, and CA 19-9 for pancreatic cancer [10]. However, the correlation between the presence of biomarkers and an increased risk of developing cancer remains still difficult to assess. In addition, a great number of potential predictive tumour markers have not yet a defined clinical significance. This is largely due to the lack of methods that are both ultrasensitive, simple, and easily transferable to the clinical use. Diverse methods are currently applied to the quantitative and qualitative detection of tumour markers [11], but all these methods present diverse limitations in terms of sensitivity, such as enzyme-linked immunosorbent assay (ELISA) [12], colorimetric assay [13], and electrochemiluminescent assay, and in terms of complexity and/or costs, such as surface plasmon resonance [14], electrochemical assay [15], and mass spectrometry. Mass spectrometry coupled with liquid chromatography, for instance, is currently one of the most powerful tools that combines high sensitivity and mass accuracy to deal with the challenges impose by proteome complexity. However, its clinical use is limited by the complexity and time consuming of the method that requires multiple analysis steps to fully resolve single proteins or peptides. Moreover, the uncertainty elicited by non-tumour-associated changes in plasma and serum proteins requires the study of panels of proteins to completely profile cancer-associated markers. This constitutes another barrier that is only partially obviated by flanking these methods with protein-capture-based microarrays [16].

The development of new methodologies to surmount these limits is crucial. In this work, we exploited the use of surface-enhanced Raman scattering (SERS) for in vitro characterization and ultrasensitive quantitation of tumour markers [17–19]. This couples the power of Raman spectroscopy in molecule identification with the drastic improvement of the signal mediated by plasmonic materials (e.g. gold, silver) that enhances Raman signal by a factor up to 10^15^ [19, 20] allowing the detection of trace analytes. Despite the great advantages offered by SERS in terms of sensitivity, most of the research in this field suffers of some limitation. Indeed, typically to accomplish SERS, the use of Au or Ag colloid nanoparticles (NPs) is exploited. Diverse authors reported the use of NPs for effective SERS detection of tumour markers [21]. However, the strong tendency of NPs to form aggregates could affect results, making them difficult to interpret and hardly reproducible [22, 23]. On the other hand, most of the SERS immunoassays that use nanostructured solid surfaces employ sandwich-like methods [24–26] that lead to an overly complex and time-consuming procedure. To overcome the limits imposed by NPs and to simplify the commonly used sandwich-like methods, we developed an easy, fast, and ultrasensitive SERS-based system exploiting the development of antigen-capture nanostructured gold solid surfaces suitable for a direct immuno-SERS approach. The antigen-capture SERS system was made following a two-step functionalization procedure for the covalent immobilization of anti-human LGALS3BP antibodies on gold SERS solid substrates. Galectin-3-binding protein (LGALS3BP) also known as 90K is involved in tumour growth and progression and was chosen as a model of a tumour marker since it has been found elevated in serum of patients with different types of cancer, such as breast, prostate, and colorectal cancers [27–30].

High levels of 90K are associated with the occurrence of metastasis and with an increased resistance to chemotherapy [31, 32]. We first used a recombinant 90K protein (R56) to validate the detection system by individuating the limit of SERS detection (LOD), and then, we extended the study to the analysis of sera from oncologic and healthy donors. Interestingly, the system was effective both for ultrasensitive quantitative and qualitative analysis and for the characterization and distinction of samples with different biochemical compositions, allowing a direct analysis of complex biological fluids such as serum.

## Materials and methods

### SERS substrates

We used the “MatoS” substrates (AtoID^™^) as the SERS platform. They are made from silicate glass with an overall volume of 12.5 × 5 × 1 mm^3^ and an active area of 3 × 5 mm^2^. A thin gold layer is deposited on the glass surface for an optimized excitation wavelength from 600 nm to NIR. The active area is composed of stochastic nanostructure feature fragments from tens of nanometres to few microns, and such variety meets resonance criteria for many different vibrational modes of molecules.

### Procedure of functionalization

The MatoS substrates were subjected to a two-step functionalization process to covalently bind antibodies to the gold surface that consisted of a first binding with a linker molecule (Nanocs) followed by the attachment of the capture antibody, as described below.

### Attachment of linker molecule

MatoS were first functionalized with a lipoic acid/maleimide heterobifunctional linker (LA-dPEG3-Mal, Nanocs); then, the MatoS substrates were immersed in a 4 mM anhydrous ethanol solution of LA-dPEG3-Mal linker molecule and gently stirred for 30 h at room temperature in dark. After incubation, the functionalized substrates were washed with anhydrous ethanol to remove the unbound linker.

### Antibody preparation

Murine Humanized Anti-Human 90K/LGAL3SBP antibody (1959Cr) (MediaPharma) was solubilized in binding buffer (10 mM sodium phosphate, 0.15 M sodium chloride, pH 6.6) at a final concentration of 1.45 μM.

### Antibody reduction

Tris(2-carboxyethyl) phosphine (TCEP) from Sigma-Aldrich was added to the antibody solution to the final concentration of 1 mM and incubated for 2 h at 37 °C, to partially reduce antibody cysteines.

### Antibody binding to SERS substrates

LA-dPEG3-Mal-functionalized MatoS were then incubated with reduced or non-reduced antibody solutions and stirred for 2 h at room temperature. After incubation, 1959Cr-functionalized MatoS were washed with deionized water to remove free antibodies and air-dried. MatoS were stored in dark up to analyses.

### ELISA assay

Serum levels of 90K were first measured by sandwich ELISA. Murine anti-Human 90K/LGAL3SBP (SP2) and 1959Cr antibodies (MediaPharma) were used as capture and detection antibodies, respectively; LGALS3BP (R56, Media Pharma) was used as standard antigen for calibration curve.

Briefly, sandwich ELISA assays were carried out on 96-well microplates (Falcon) as follows: microplate wells were coated with 100 μl of 1 μg/ml SP2 antibody, dissolved in phosphate-buffered saline (PBS), and incubated overnight at 4 °C under stirring. Blocking was carried out with 200 μl/well of blocking solution (PBS, bovine serum albumin 0.5%) for 1 h at room temperature. Calibration curve was carried out with 1000 to 15.6 ng/ml of R56 standard antigen. Serum samples from oncologic and healthy donors were diluted 1:1000 in blocking solution. Washing steps were performed in PBS in presence of 0.05% Tween. Anti-human IgG HRP conjugate antibody (Sigma-Aldrich) was used for quantitation, and TMB supersensitive liquid substrate (Sigma-Aldrich) was used for peroxidase detection. Measures were performed using a microplate reader (Tecan Spark).

### Atomic force microscopy

Both unfunctionalized and LA-dPEG3-Mal- and 1959Cr-functionalized MatoS substrates were characterized by atomic force microscopy (AFM) using a Bruker Dimension Icon (Bruker, Santa Barbara, CA, USA) microscope in standard tapping mode and under ambient conditions. The measurements were carried out with Bruker super sharp TESP-SS cantilevers with a resonance frequency of about 320 kHz and tips with a radius of curvature < 5 nm. Image analysis was performed with the Gwyddion 2.5 software. Except for flattening, no image post-processing procedure was applied. The surface roughness (Rp) was evaluated over 1.5 × 1.5 μm^2^ images as the root-mean-square (RMS) average of height deviation taken from the mean image data plane.

### SERS spectroscopy

SERS measurements were carried out using a BW&Tek i-Raman spectrometer (BW&Tek), equipped with a GaAlAs diode laser emitting at 785 nm, with adjustable power up to 300 mW. The laser beam was focussed on the sample through an optical microscope with × 40 objective (N.A. 0.5), in a spot of 40 μm diameter. The spectra were acquired by exciting the sample with 90 mW laser power and 90 s acquisition time. For each substrate, we performed the measurements on five different zones of the active area, with five repetitions. Spectra were collected in three successive steps: (1) on the clean substrates (empty-MatoS), to verify the homogeneity of the substrates and check the absence of remarkable spectral features on the clean active area; (2) after the functionalization process, to check the homogeneity of the functionalized area; and (3) in presence of the 90K antigen, to highlight its spectral contribution.

After being acquired, SERS spectra were processed through the following steps: (1) cropping of the spectral region between 350 and 1700 cm^−1^ with resulting datasets of 742 channels (pixels); (2) background fluorescence subtraction; (3) spike removal; (4) normalization between 0 and 1 of the residual signal. These steps were performed by processing the raw spectra applying a home-developed routine with an iterative procedure according to Zhao et al. [33] (see Electronic Supplementary Material (ESM) Figs. S1 and S2 for further details).

The SERS spectra were analyzed by using principal component analysis (PCA) [34–36] which is a multivariate technique that is performed on different datasets (in the case of this manuscript, the SERS spectra from different classes like spectra from the clean SERS substrate, spectra from the functionalized substrate, spectra from the substrate with antigen) to understand the factors affecting the spectral variation across the samples when these are not clearly recognizable because of the low concentration of the analytes. PCA is calculated from the covariance matrix of the original dataset (containing spectra from different groups), and it can be considered like an axis rotation transformation that calculates a new set of axes, called principal components (PCs), that enhances the maximal directions of variance within the dataset. Each spectrum is then projected in this new *n*-dimensional space of PCs and can be visually represented by the first two or three PCs (typically containing the larger fraction of variance in the dataset). In this way, the dimensionality of the spectrum is reduced into two or three coordinates in the 2D or 3D PC space and it can be typically represented by a point in this space. In Raman-SERS spectroscopy, this method enhances the weak spectral differences between different classes of spectra in such a way that, if the spectra of one group (for example all the spectra from the clean substrate) exhibit common spectral features, they will be grouped in a well-defined region of the PC space creating well-defined clusters immediately recognizable. An alternative discriminant analysis method that would efficiently classify samples with different levels of 90K could be PLSR (partial least squares regression), but PCA was here preferred because we focussed on relatively few intermediate concentrations of 90K.

### Sample preparation for 90K SERS detection

Recombinant 90K (R56) and serum samples were diluted in PBS at different concentrations starting from 0.5 μg/ml for R56, in presence or absence of 1% serum bovine albumin (BSA, Euroclone). In the case of serum samples, all sera were subjected to a 10^3^, 10^6^, and 10^9^ factor dilutions before analyses. 1959Cr-MatoS were immersed in 300 μl of recombinant 90K or in previously diluted serum samples (as such or diluted in phosphate buffer) and incubated at different times (from 5 to 60 min) at room temperature. After incubation, 90K-1959Cr-MatoS samples were intensively washed with double distilled water to remove unbound ligands and air-dried before SERS analysis.

## Results

### SERS substrate functionalization

We developed a SERS detection system based on antibody-functionalized solid nanostructured SERS gold surfaces. SERS gold-coated MatoS substrates were used since they are made from silicate glass that is a low-cost and very stable material rendering them suitable for routine analyses and medical diagnostics.

**Fig. 1:**
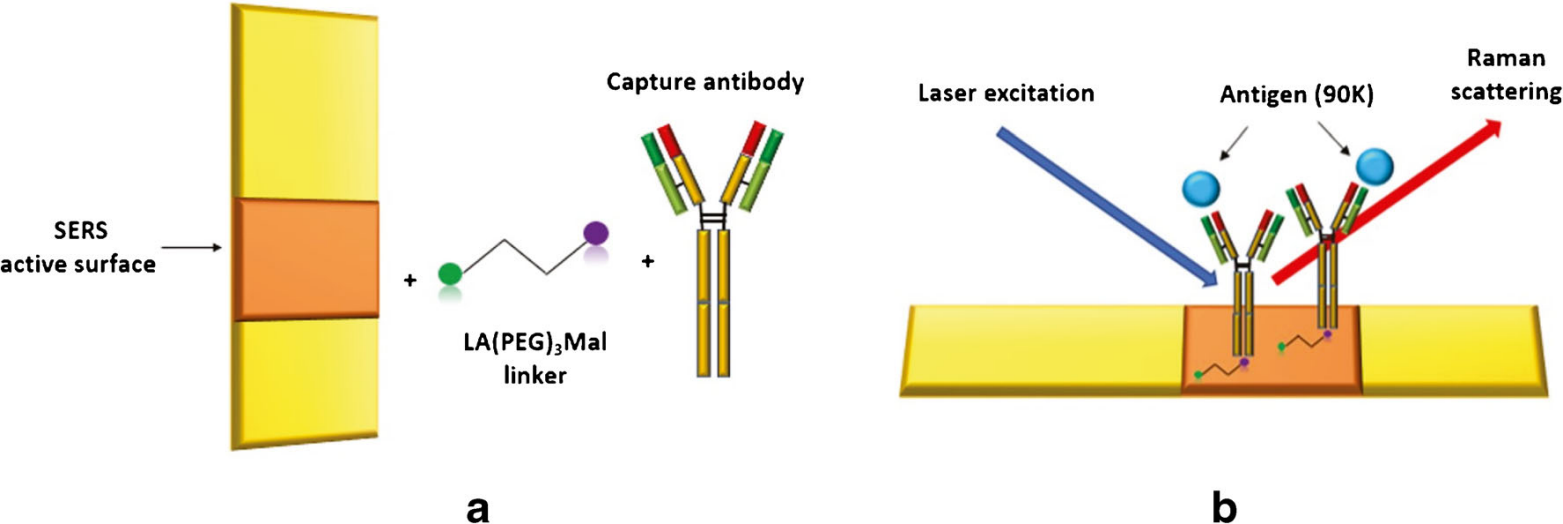
Two-step functionalization of SERS substrates. **a** A heterobifunctional linker mediates the covalent binding of anti-90K capture antibody to the gold SERS active surface of nanostructured substrates; the active SERS surface (in orange) is made of stochastic nanostructure feature fragments from tens of nanometres to few microns nanostructured gold. **b** Schematic representation of Raman scattering after the interaction of the system with the 90K antigen

We established a protocol to covalently bind antibodies to gold nanostructured solid surfaces by using sulfhydryl-reactive cross-linker chemistry. This was made through a two-step process involving the use of a heterobifunctional linker (LA-dPEG3-Mal) constituted of lipoic acid and maleimide. Lipoic acid was used to mediate the binding with the gold surface due to its high affinity for metals, and maleimide was chosen to mediate the covalent binding with antibody free cysteines (Fig. 1). Indeed, compared with the most used amine-reactive chemistry, maleimide chemistry allows numerous advantages such as a more specific and directional binding of antibodies and does not interfere with the functionality or exposure of antigen binding site. Following the functionalization of MatoS (empty-MatoS) with the heterobifunctional linker, linker-MatoS were thus functionalized with the Murine Humanized Anti-Human 90K 1959Cr antibody (1959Cr-MatoS).

**Fig. 2:**
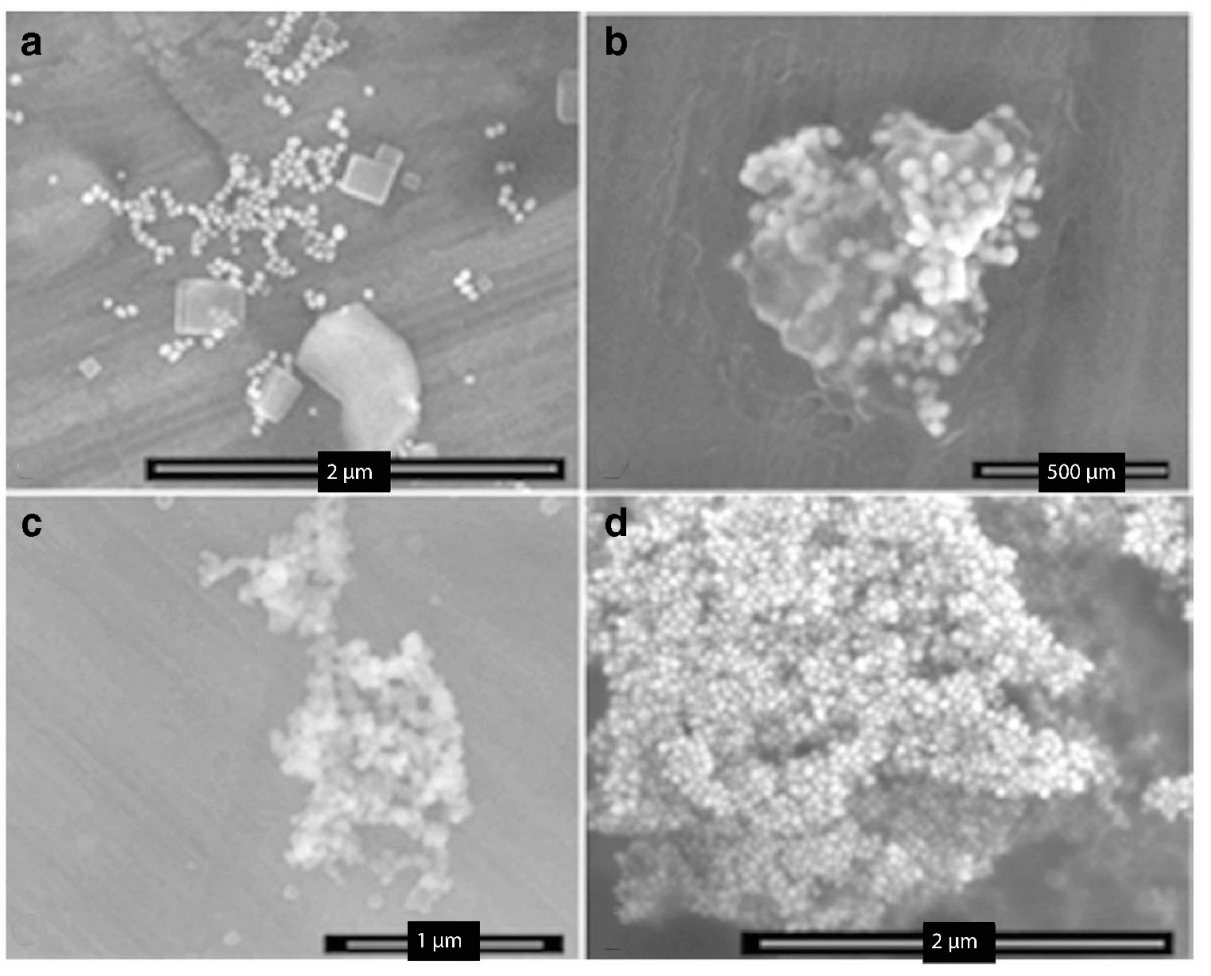
SEM images show the different levels of aggregation of AuNPs depending on the functionalization state. **a** Non-functionalized AuNPs. **b** LA(PEG)3Mal AuNPs. **c**, **d** Anti-90K capture (1959cr) antibody-functionalized AuNPs

The choice of using nanostructured supports instead of nanoparticles lied on the attempt to obtain stronger and higher reproducible results. Indeed, when a similar protocol of functionalization was applied to gold nanoparticles (AuNPs), we observed a high level of AuNP aggregation, depending on their state of functionalization that significantly affects experimental reproducibility and could easily generate artefacts (Fig. 2).

### Characterization of functionalized SERS substrates

#### Atomic force microscopy analyses

The functionalization procedure was monitored after each step by atomic force microscopy (AFM) and Raman spectroscopy. Empty-MatoS, linker-MatoS, and 1959Cr-MatoS were first characterized by AFM (see ESM Fig. S3). AFM imaging of empty-MatoS (ESM Fig. S3a) showed AuNPs with an average diameter of 120 nm which are partially aggregated; the resulting RMS roughness of the surface is 200 nm. Compared with this reference surface, linker-MatoS (ESM Fig. S3b) showed a much shallower surface morphology (Rp is reduced by almost a factor of 10, to about 20 nm), well matching the presence of a top molecule film which smoothens out the NP roughness. Conversely, the AFM analysis shows a larger roughness for 1959Cr-MatoS antibody samples (ESM Figs. S3c and S3d), Rp being 210 nm and 150 nm for the reduced and non-reduced antibody, respectively. This is due to the formation of aggregates which, for the reduced sample, have less isotropic and more structured shapes, with the presence of columnar structures reaching heights of about 500 nm.

#### Raman spectroscopy analyses

**Fig. 3:**
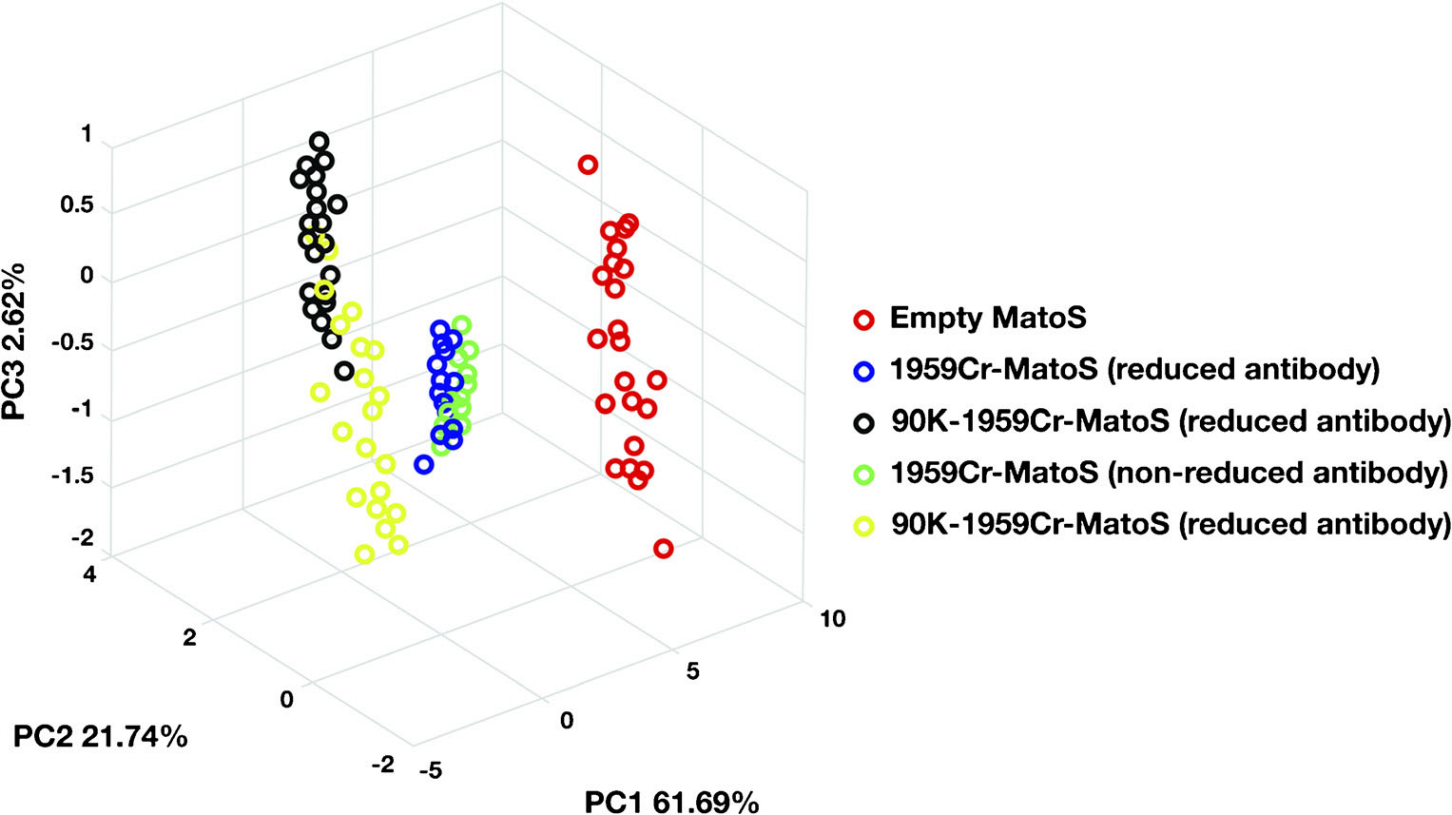
PCA of Raman spectra from substrates analyzed before and after antibody functionalization and in presence of the 90K antigen. PCA data shows a clear separation between empty-Matos, 1959Cr-MatoS, and 90K-1959Cr-MatoS

The functionalization of MatoS substrates was also confirmed by Raman spectroscopy (see ESM Figs. S4 and S5). Raman spectra were collected, and chemometrics analyses by using principal component analysis (PCA) were performed to individuate differences and/or common features between samples. PCA results showed a significant clustering between empty-MatoS and functionalized-MatoS (Fig. 3) demonstrating a surface diversity induced by functionalization. In contrast, there were no significant differences between diverse empty-MatoS confirming their surface homogeneity, thus excluding spectral interferences due to SERS substrates.

Raman analyses were also used to identify the best conditions of functionalization. Different concentrations of linker and of reduced or non-reduced antibody were used to functionalize substrates. As result, we selected 4 mM and 1.45 μM for linker and reduced antibody, respectively, that gave the best PCA clustering profile (data shown in ESM Fig. S6).

### Immuno-SERS for 90K detection

#### System validation

We used a direct immuno-SERS to detect and characterize 90K protein. We first used a recombinant 90K protein (R56) as a standard to evaluate antigen/antibody interaction and to define the LOD.

1959Cr-MatoS were incubated with different known concentrations of recombinant 90K, starting from 0.5 μg/ml that is a good detectable concentration through ELISA assay.

To individuate the optimal incubation time, the interaction antigen/antibody in a range from 5 to 60 min was evaluated. After incubation, we performed intensive washes with double distilled water to completely remove non-specifically bound and unbound 90K antigen.

**Fig. 4:**
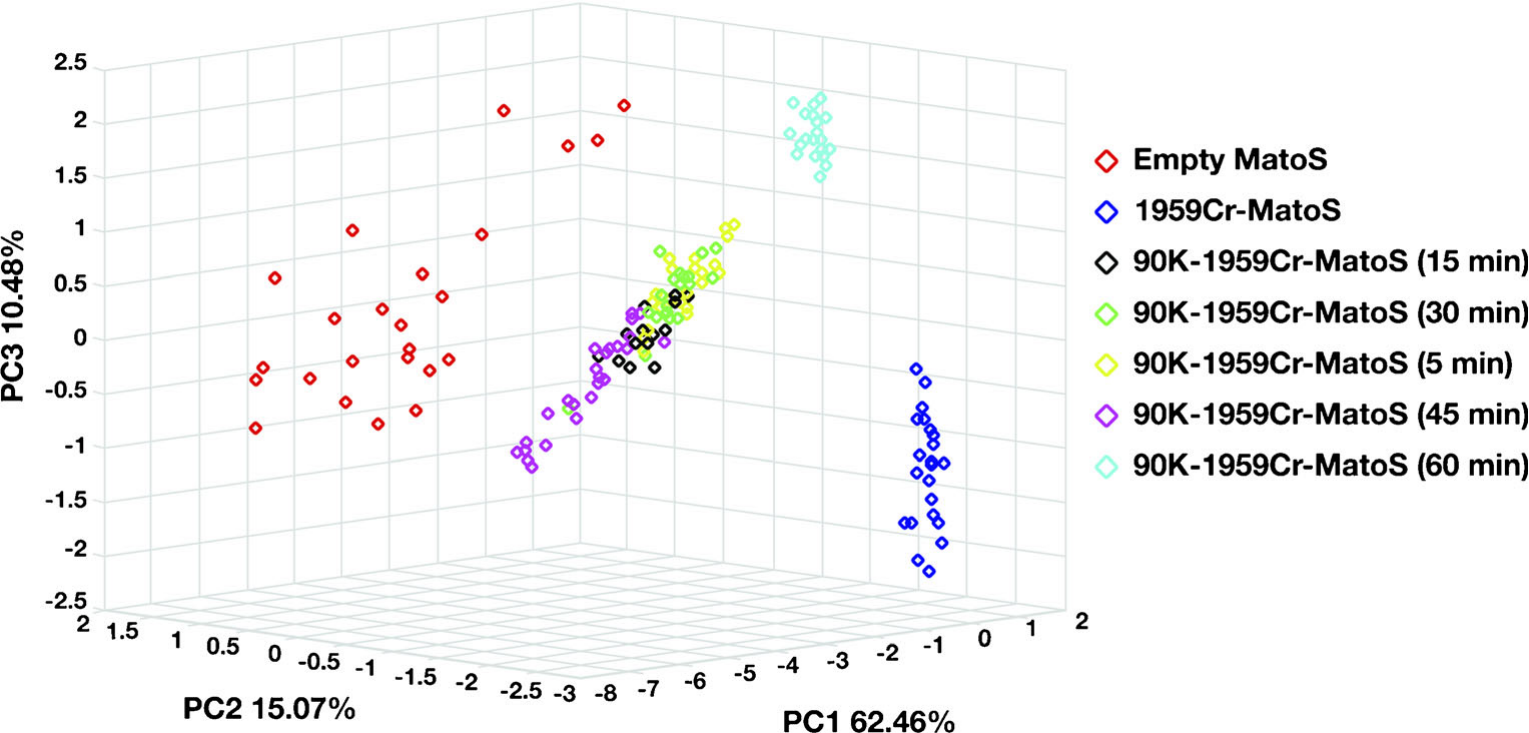
PCA of Raman spectra at diverse time of 90K antigen exposure. There is a clear separation between 1959Cr-Matos and 90K-1959Cr-Matos already after 5 min of incubation with the antigen

PCA of Raman spectra showed a clear clustering between 1959Cr-MatoS and 90K-1959Cr-MatoS as early as 5 min of incubation, indicating the efficiency of antigen/antibody interaction even at relatively short times. However, from comparing PCA data of 90K-1959Cr-MatoS at different times of antigen exposure, we observed that data became gradually more defined with the increase of the exposure time (from 15 to 45 min) until a stronger definition was achieved at 60 min (Fig. 4). We concluded that 15 to 30 min of exposure were adequate to obtain a fast and efficient 90K detection.

#### Detection of 90K in serum samples

The detection of 90K on serum samples from oncologic and healthy donors to assess the efficiency of the detection system in a complex fluid was carried out on serum samples that were heterogeneous for tumour type and donor age. 90K levels of serum samples from both healthy and oncologic donors were first analyzed through ELISA, a conventional method of routine clinical use that was used as a referring method, and the LOD was assessed to be around 15 ng/ml. ELISA results indicated the presence of 90K in all the analyzed samples. As expected, 90K levels were notably higher in oncologic sera (average concentration was 0.5 μg/μl) than in healthy sera (average concentration was 90 ng/μl).

**Fig. 5:**
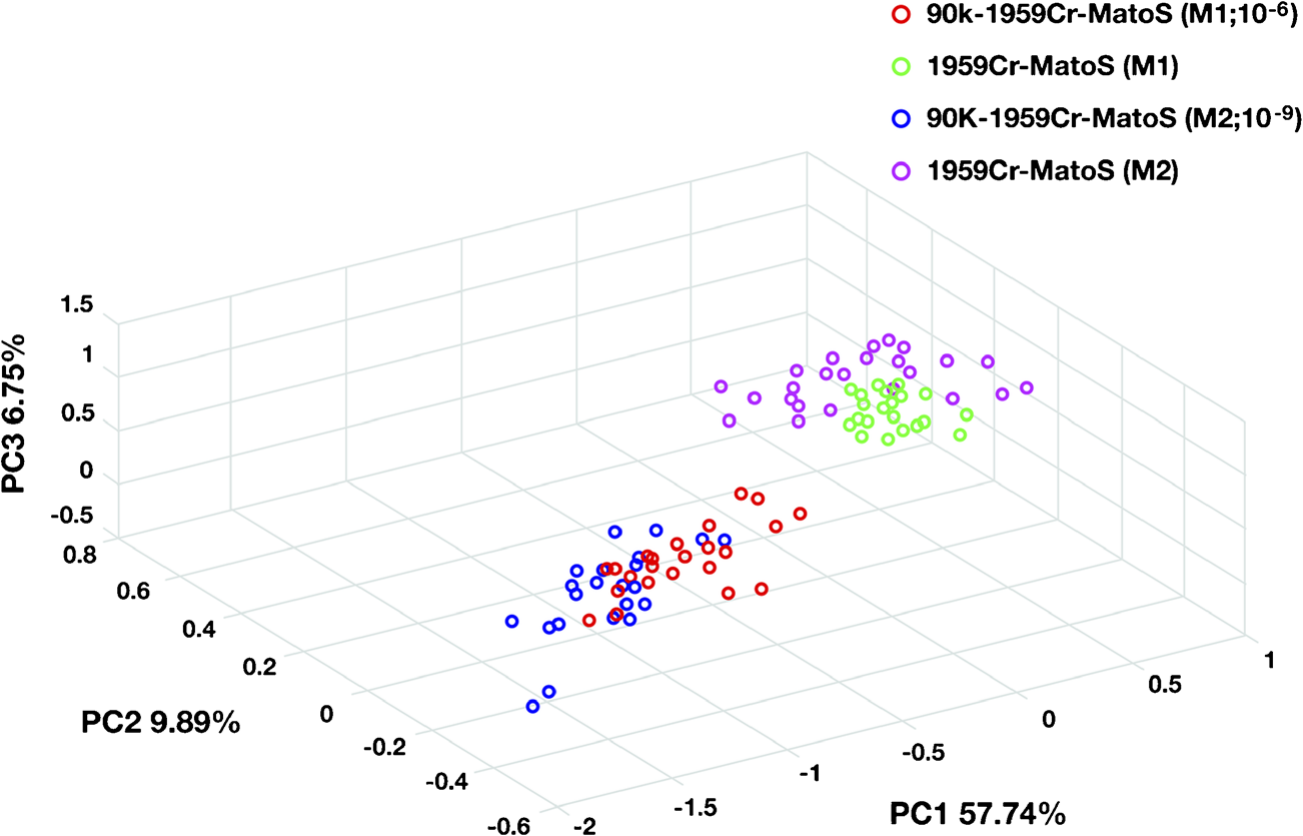
PCA of Raman spectra of oncologic serum samples. Data refers to 1919Cr-MatoS analyzed before the interaction with oncologic sera (M1 and M2) and after the incubation with two diverse serum dilutions (M1; 10^−6^) and (M2; 10^−9^)

SERS analyses of serum samples were made by diluting sera of 10^3^, 10^6^, and 10^9^ factors. Incubation time for antibody/antigen interaction was 30 min. As results, PCA data showed a clear separation between 1959Cr-MatoS and 90K-1959Cr-MatoS from oncologic serum samples. Data from PCA indicated a clear separation of 1959Cr-MatoS and 90K-1959Cr-MatoS up to 10-9 cancer serum dilution (10^−12^ g/ml), demonstrating that the system was effective for picogramme detection of the 90K antigen (Fig. 5).

**Fig. 6:**
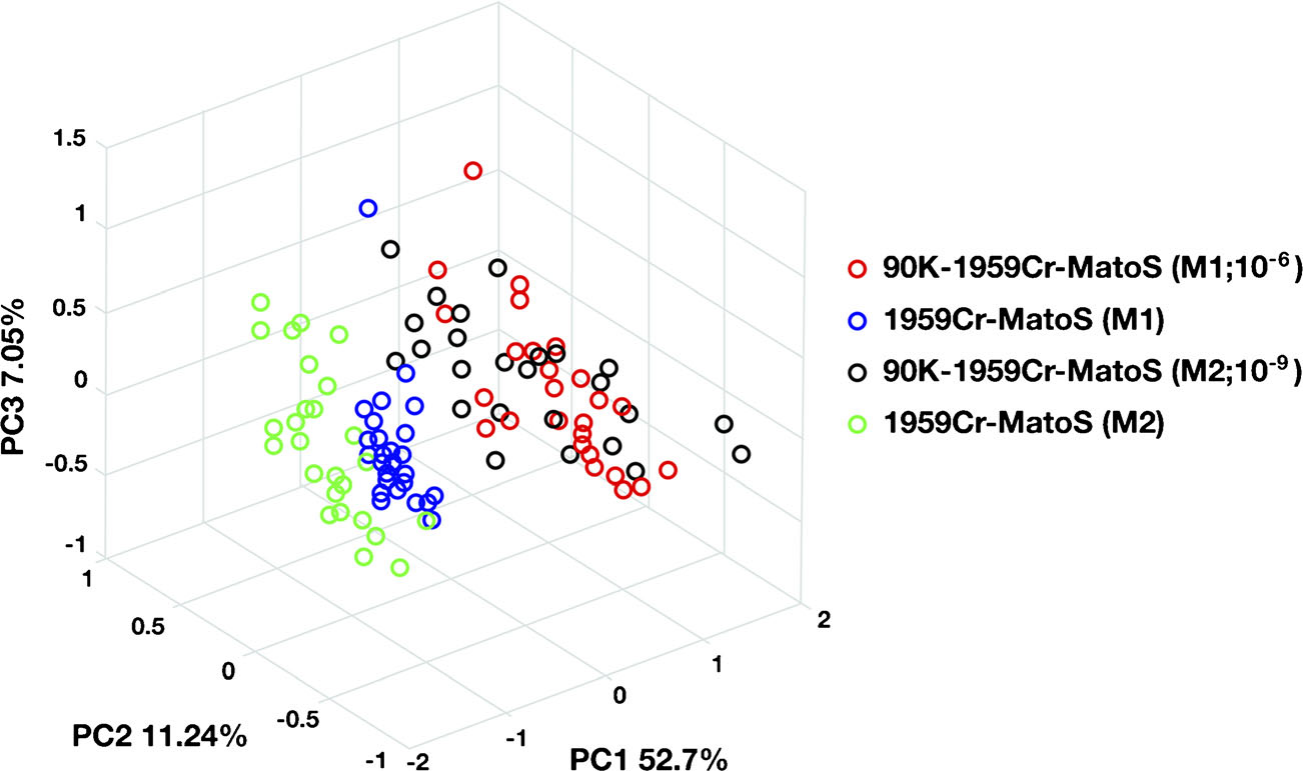
PCA of Raman spectra of healthy serum samples. Data shows the lack of a clear separation between 1959Cr-MatoS before and after the incubation with two diverse healthy serum dilutions, indicating that the presence of other serum components does not overlap with 90K detection

Importantly, data confirmed that the system can detect antigen/antibody interaction in complex fluids (e.g. serum) without be subjected to interferences from other serum components. This last aspect is confirmed by the lack of a clear clustering between 1959Cr-MatoS and 90K-1959Cr-MatoS of healthy serum samples (Fig. 6). Indeed, healthy sera have lowest levels of 90K but should contain similar amount of other serum components. Due to the variability of 90K concentration in healthy sera (such as in cancer sera), healthy sera with the lowest 90K level (not detectable with ELISA assay at 10^3^ dilution) were selected as control.

## Discussion

Due to its high performance and specificity (use of antibodies), immuno-SERS attracted the attention of several research groups in the attempt of detecting trace of analytes in complex fluids [37]. However, most of the developed immuno-SERS methods make use of nanoparticles that present numerous disadvantages: the strong tendency of nanoparticles to form aggregates and importantly the high complexity in synthesis processes and management are indeed not in line with clinical applications. Several works considered the use of solid nanostructured supports that gives highly reproducible results [37]. However, for immuno-SERS applications, solid substrates need to be functionalized with capture antibodies, and the type of functionalization procedure requires to be carefully calibrated depending on diverse factors such as the type of SERS substrate, the nature of the analyte, and the matrix to analyze.

In this work, we developed a simple and efficient protocol of functionalizing SERS gold substrates with capture antibodies for serum detection of the 90K biomarker. Importantly, we opted for the covalent immobilization of antibodies on gold SERS active surfaces by using maleimide chemistry: the maleimide group forms a covalent thioether linkage by reacting with the thiols of antibody free cysteines (thiol-Michael addition) [38, 39]. The functionalization of gold solid substrates with maleimide was performed using a heterobifunctional linker containing both maleimide and lipoic acid that was used to allow the binding of the linker with gold. The maleimide functionalization method was specifically assessed for gold solid substrates starting from the work that Mattoussi and colleagues carried out on AuNPs [40]. Indeed, the use of a linker molecule simplifies greatly the process of gold maleimide functionalization that is normally performed by more complex chemical methods [41]. The use of maleimide as a functional group and the consequent covalent immobilization of antibodies induce several advantages, with respect to other functional groups (i.e. groups that mediate amine-reactive chemistry) [42] such as a more controlled and directional binding of antibodies to the gold surface that ensures the exposure of the antigen binding site, and a strong interaction of the capture antibody with SERS substrates that is a crucial requirement for the analysis of complex matrices such as serum. We also investigated on the stability of thiol-maleimide conjugates in our experimental conditions since some authors reported issues, even if mainly related to in vivo applications [43]. We demonstrated that the conjugated MatoS substrates are suitable for long-term storage giving reproducible results up to several months. Finally, this procedure made use of a linker molecule that is often used for SERS applications [44]. In this work, the average distance of the SERS surface from the sites binding the antigen is evaluated to be ≤ 10–14 nm (complex linker/antibody) that is consistent with a significant SERS effect [45, 46].

For detection analyses, we exploited the use of a simplified direct immuno-SERS method that allowed a one-step analysis of the antibody/antigen interaction by directly incubating the antibody-functionalized SERS substrates with the analyte samples. Although some authors applied a similar direct immuno-SERS for analyte detection [47], most of the commonly used immuno-SERS methods are sandwich-like [48] and require more than one-step analysis, thus with a higher complexity and time consuming.

Beyond its simplicity, the detection procedure was revealed to be ultrasensitive, allowing to detect picogramme traces of the 90K biomarker in serum samples (LOD was 10^−12^ g/ml), fast (exposure time was ˂ 30 min), and able to discriminate between samples with different biochemical composition. Furthermore, the system was suitable for the detection of the 90K antigen in complex fluids such as serum samples and without suffering from interferences of other serum components.

## Conclusions

In this work, we developed a method of functionalization of nanostructured gold solid SERS substrates to covalently immobilize antibodies and demonstrated the efficiency of a direct immuno-SERS procedure for the 90K antigen SERS detection. We found that the developed detection system has proven effective for characterizing the 90K antigen and discriminating against different samples for biochemical composition. Due to its versatility (the functionalization procedure could be effectively applied to diverse antibodies), we aim to extend this work to the study of panel biomarkers by developing multiplexed antibody-functionalized SERS substrates, as a high-throughput tool for ultrasensitive biomarker detection and validation. This, coupled to the collection of a significant number of heterogenic serum sample analyses for clinical correlations, could provide the foundation for the development of a medical diagnostic device with a high predictive power that overcomes the proteome complexity of biological fluids and is easily transferable to the clinical use.

## Electronic supplementary material

ESM 1 (PDF 1.28 mb)
