# Supplementary material for: Surface-enhanced Raman scattering (SERS)–based immunosystem for ultrasensitive detection of the 90K biomarker
Source: Anal Bioanal Chem. 2020 Sep 2;412(27):7659–67. doi: 10.1007/s00216-020-02903-2 (PMC7533257; doi:10.1007/s00216-020-02903-2)
Supplement: Supplementary file 1 — (PDF 1.28 mb) [file 216_2020_2903_MOESM1_ESM.pdf]

Analytical and Bioanalytical Chemistry

Electronic Supplementary Material

Surface-enhanced Raman scattering (SERS)-based immunosystem for ultrasensitive detection of the 90K biomarker

Valentina Gallo, Antonia Lai, Alessandra Pasquo, Salvatore Almaviva, Stefano Iacobelli, Luca Persichetti, Giovanni Capellini, Giovanni Antonini

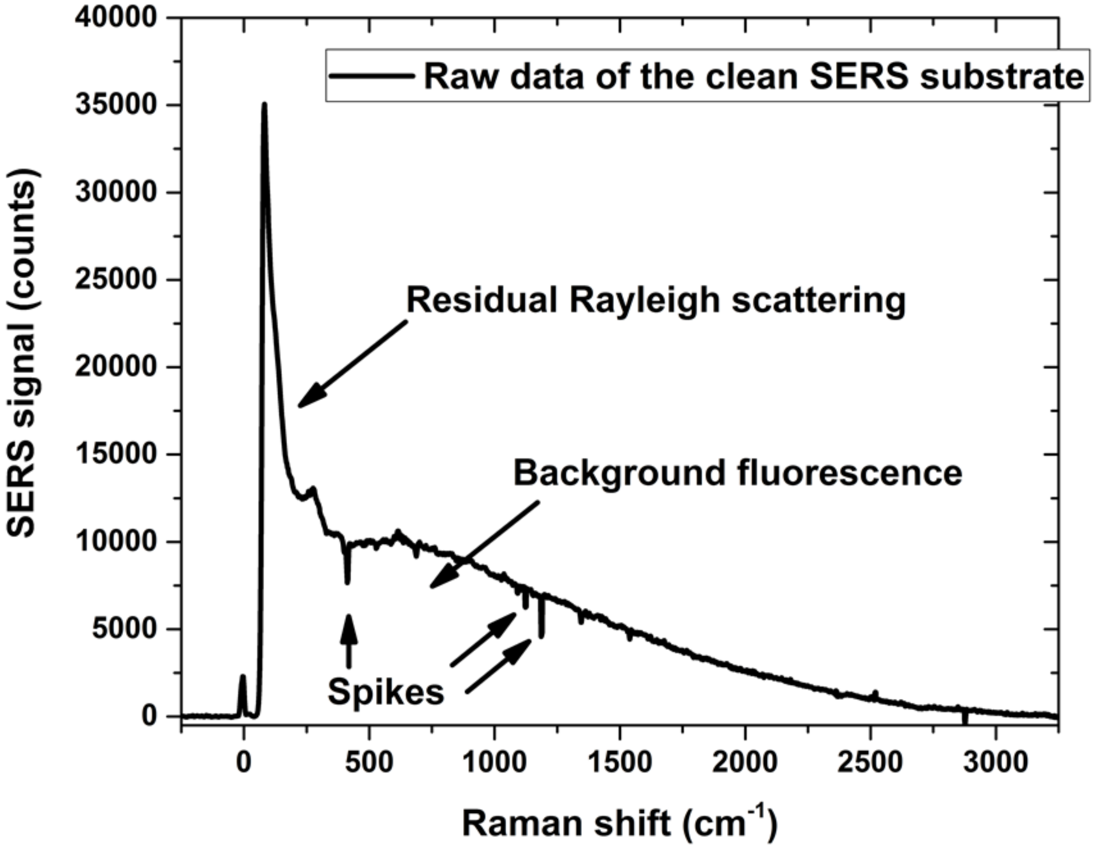

Fig. S1 Typical SERS spectrum with highlighted some unwanted features

The raw SERS-Raman spectra are affected by unwanted features and components that prevent the analysis of the meaningful part of the spectrum, so typically a spectra processing is needed.

In the case of the spectra acquired for this manuscript the raw signal typically appears as reported in the figure S1 (this is an example of a clean SERS substrate).

The unwanted components to remove are 1) the residual Rayleigh scattering; 2) random spikes; 3) the background fluorescence that is often present in a Raman spectrum. Then, for the sake of uniformity, the residual signal is normalized in the interval [0;1] ready to be processed with statistical methods (e.g. Principal Components Analysis).

1) The residual Rayleigh signal is removed by cropping the spectrum from 350 cm-1 as reported in the manuscript.

2) The spikes are removed by applying to the residual signal the MATLAB™ spikes removal function “medfilt1” [1] that replaces every point of a signal by the median of that point and a specified number (5, in the case of the present spectra) of neighbouring points.

3) The background fluorescence is removed by applying a recursive procedure similar to that reported in Zhao et al. [2] and here briefly summarized: an iterative smoothing procedure is applied to the residual spectrum by using the MATLAB™ smoothing function “smooth” [3] and, after each iteration the raw signal and the smoothed signal are compared pixel by pixel to create a new partially smoothed spectrum. In this new spectrum the minima are retrieved from the raw signal and the rest of the signal is retrieved from the smoothed spectrum. Then, the procedure is applied again. After some iterations the result is a heavily smoothed spectrum anchored to the minima of the original spectrum with the maxima (the SERS peaks) completely levelled, ready to be subtracted to the raw signal to obtain a SERS spectrum without fluorescence background. The final result of this procedure is shown in the next figure S2, where in

the top graph the red line represents the raw cropped data, the blue line represents the result of the iterative procedure and the green line represents the raw data subtracted with the background fluorescence (i.e. green data = red data – blue data).

4) Finally, the background-subtracted data are normalized in the interval (0;1) to be uniformed for any statistical

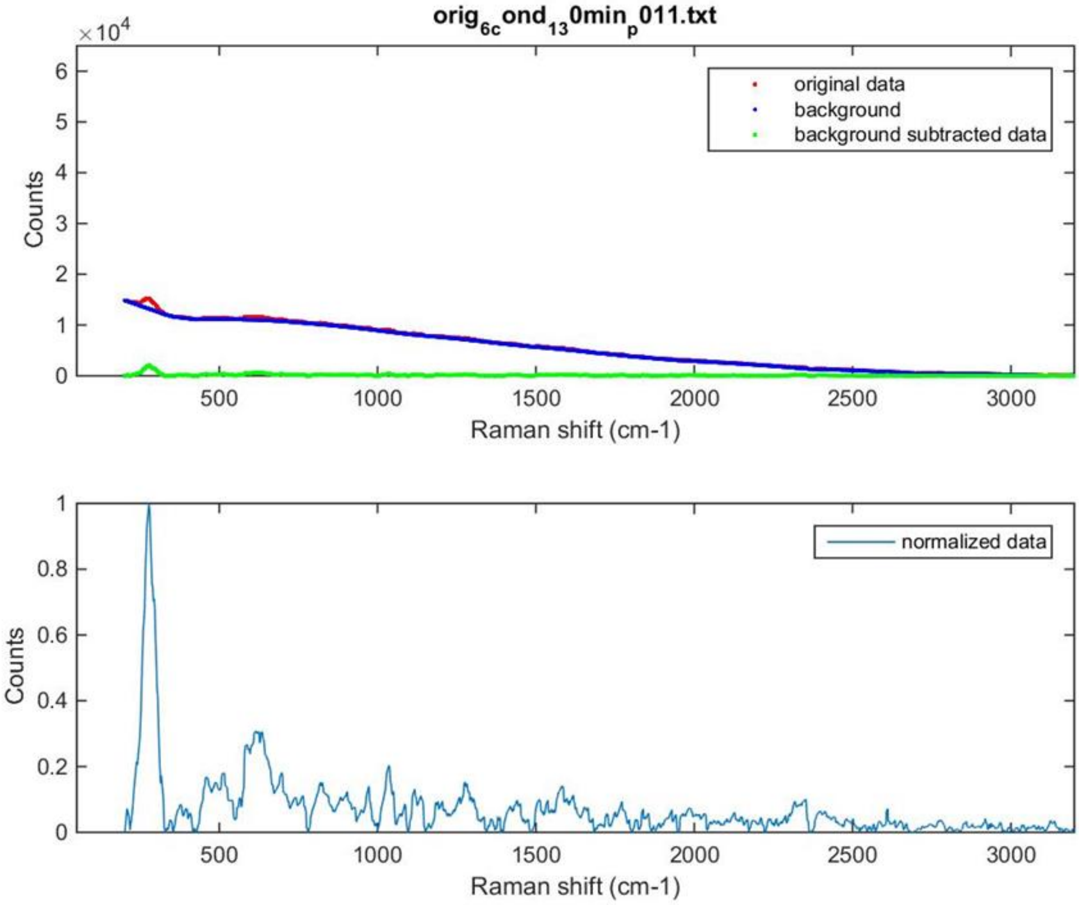

procedure, as shown in the bottom graph of figure S2.

Fig. S2 (top) the raw SERS data (red line), background fluorescence obtained after the iterative procedure described in the text (blue line), background subtracted data (red data – blue data = green data). (Bottom) green data normalized in the interval (0;1)

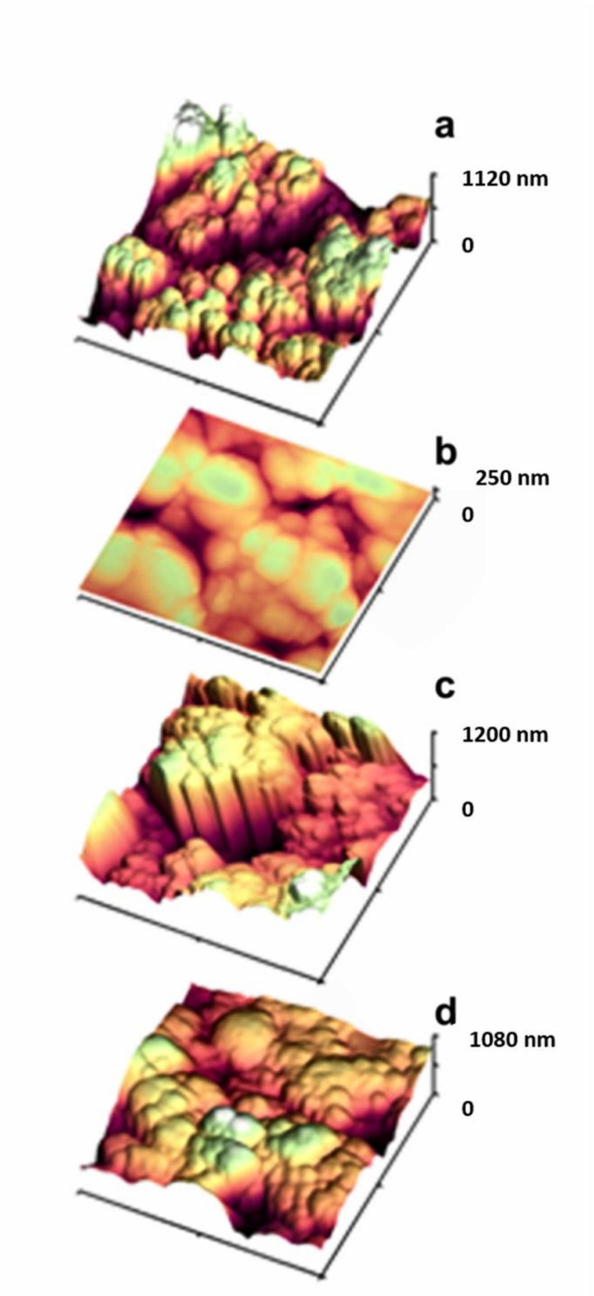

Fig. S3 3D AFM topographies showing the surface morphology of (a) empty-MatoS, (b) linker-MatoS, (c) reduced 1959Cr-MatoS antibody, (d) non-reduced 1959Cr-MatoS antibody. The height range of each panel is shown. The lateral scale is (1.5x 1.5) μm^2 for all the images

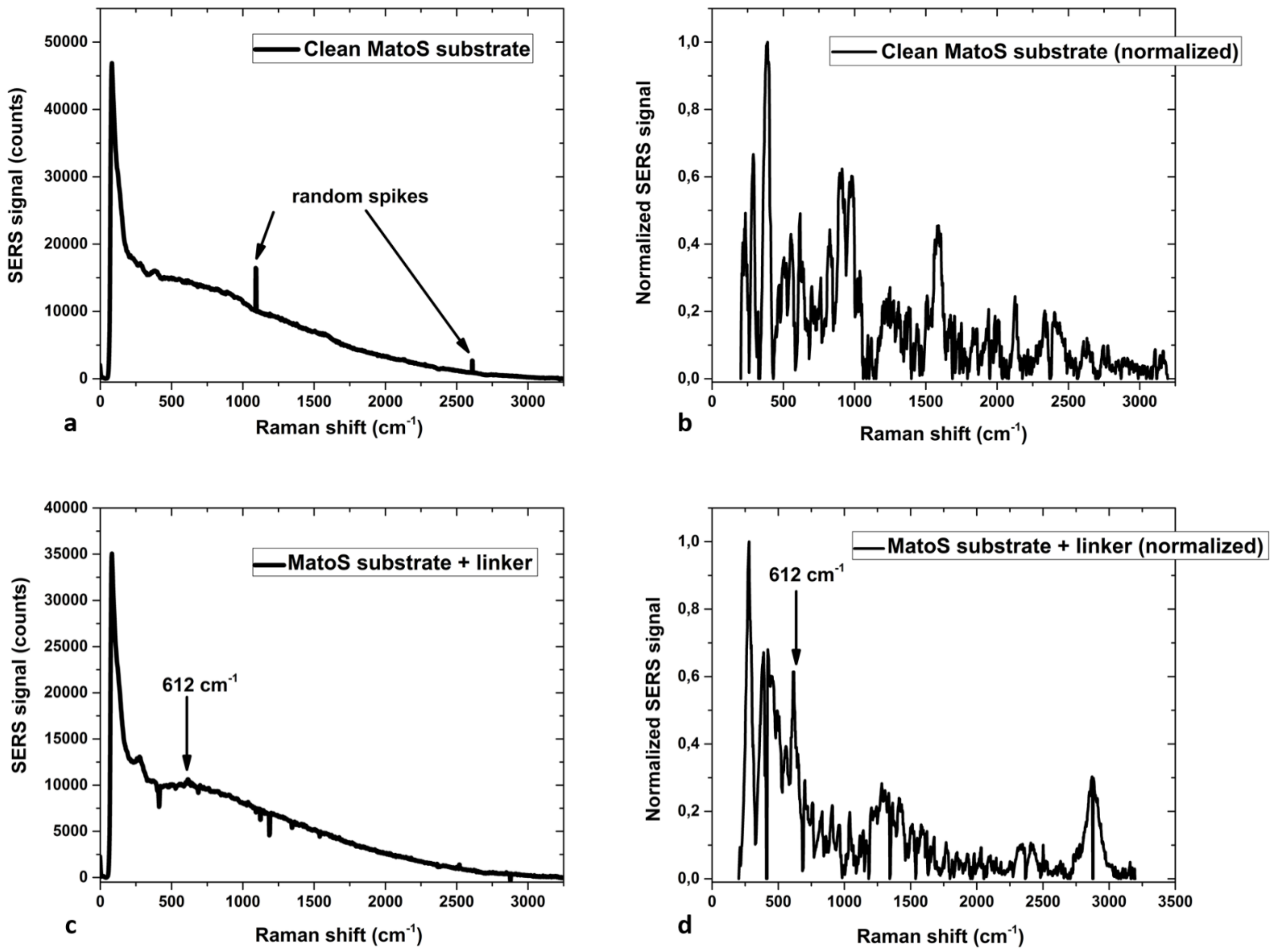

Fig. S4 a) Representative SERS spectrum of the substrate before functionalization (empty-MatoS): the spectral features are relatively flat over the fluorescence background except for random spikes; b) the previous spectrum normalized and background subtracted; c) representative SERS spectrum of the substrate after the functionalization with linker only (*linker*-MatoS): after the first step of functionalisation a spectral feature can be observed at 612 cm^{-1} that was assigned to C-S stretching of lipoic acid [4, 5]. d) spectrum of *linker*-MatoS after normalization and background subtraction

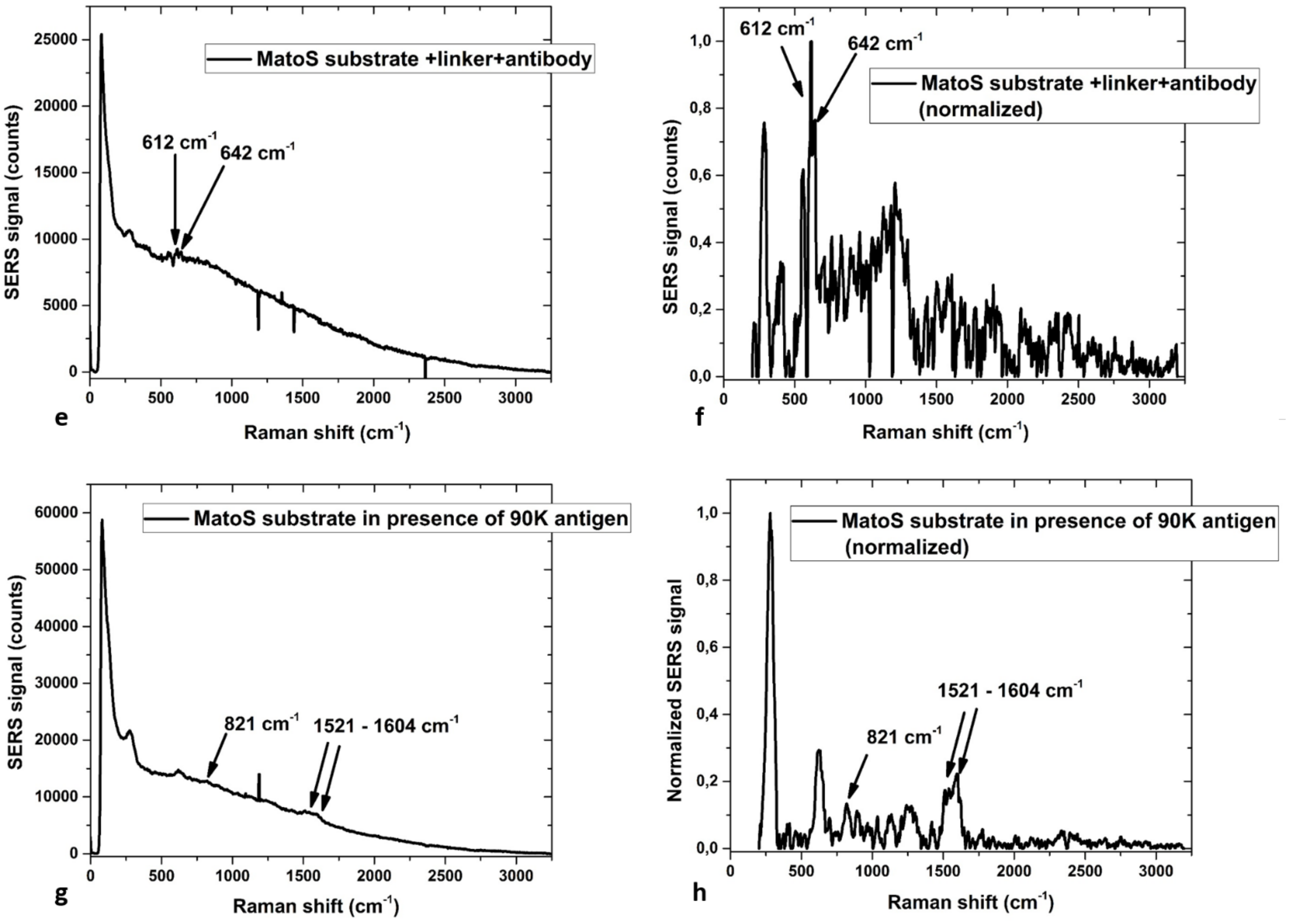

Fig. S5 e) Representative SERS spectrum of the substrate after the functionalization with linker and antibody (*1959Cr*-MatoS); two spectral features at 612 cm^{-1} and 642 cm^{-1}; f) *1959Cr*-MatoS spectrum after normalization and background subtraction; g) representative SERS spectrum in presence of the 90K antigen (*90K-1959Cr*-MatoS that shows the presence of the spectral features at 821 cm^{-1} and 1521-1604 cm^{-1}. The features at 612-640 cm^{-1} are ascribed to the previous functionalisation process. h) *90K-1959Cr*-MatoS spectrum after normalization and background subtraction. These spectral features were assigned to C-N stretching, C-C stretching and N-H bending (amide II band; 1521 cm^{-1}) and to the contribution of amino acid side chain residues such as tyrosine (642 and 821 cm^{-1}) and phenylalanine (1604 cm^{-1}) [6, 7]

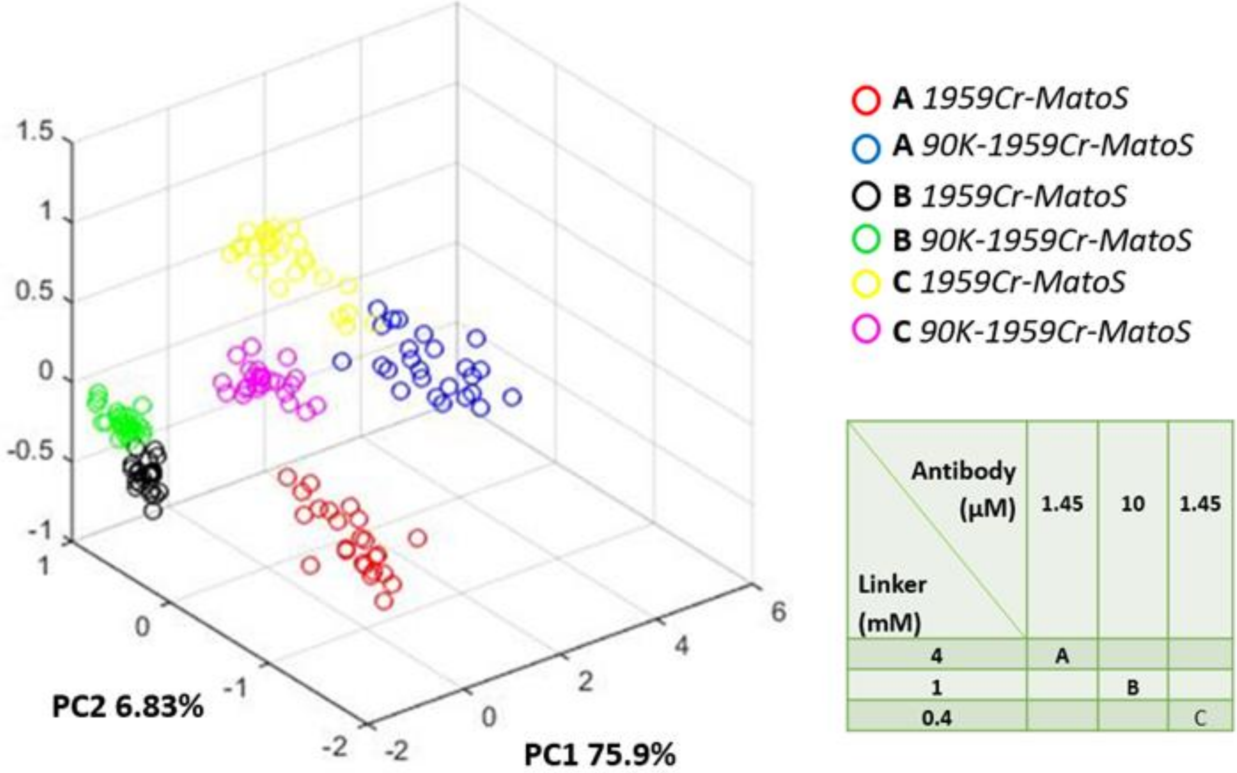

Fig. S6 PCA data from Raman spectra of MatoS substrates functionalized at diverse concentrations of linker and antibody

References

1. https://it.mathworks.com/help/signal/ug/remove-spikes-from-a-signal.html

2. Zhao J, Lui H, McLean DI, Zeng H. Automated autofluorescence background subtraction algorithm for biomedical Raman spectroscopy. Appl. Spectrosc. 2007;61(11):1225–1232.

3. https://it.mathworks.com/help/curvefit/smooth.html

4. Ikuta N, Tanaka A, Otsubo A, Ogawa N, Yamamoto H, Mizukami T, Arai S, Okuno M, Terao K, Matsugo S. Spectroscopic Studies of R(+)-α-Lipoic Acid—Cyclodextrin Complexes. Int. J. Mol. Sci. 2014;15(11): 20469-20485.

5. Lai Y, Shao H. Thioctic Acid-Modified Silver Nanoplates on Copper Foil for Low Interference Detection of Fluoranthene by Surface-Enhanced Raman Spectroscopy, Appl. Nano Mater. 2020;3(2), 2020: 1800–1807.

6. Freire PTC, Barboza FM, Lima JA, Melo FEA, Mendes Filho J. Raman Spectroscopy of Amino Acid Crystals, in Raman Spectroscopy and Applications, Kaan Maatz ed. pp. 201-224.

7. Kurouski D, Postiglione T, Deckert-Gaudig T, Volker Deckert, Ledneva IK. Amide I vibrational mode suppression in surface (SERS) and tip (TERS) enhanced Raman spectra of protein specimens Analyst. 2013; 138(6): 1665–1673. doi: 10.1039/c2an36478f.
